# Noninvasive positive pressure ventilation in unplanned extubation

**DOI:** 10.4103/1817-1737.44780

**Published:** 2009

**Authors:** Emel Eryüksel, Sait Karakurt, Turgay Çelikel

**Affiliations:** *Department of Pulmonary and Critical Care, Marmara University Hospital, Turkey*

**Keywords:** Intensive care unit, mechanical ventilation, noninvasive positive pressure ventilation, unplanned extubation

## Abstract

**BACKGROUND:**

Unplanned extubation is quite common in intensive care unit (ICU) patients receiving mechanical ventilatory support. The present study aimed to investigate the effectiveness of noninvasive positive pressure ventilation (NPPV) in patients with unplanned extubation.

**MATERIALS AND METHODS:**

A total of 15 patients (12 male, age: 57 ± 24 years, APACHE II score: 19 ± 7) monitored at the medical ICU during the year 2004 who developed unplanned extubation were included in the study. NPPV was tried in all of them following unplanned extubation. Indications for admission to the ICU were as follows: nine patients with pneumonia, three with status epilepticus, one with gastrointestinal bleeding, one with cardiogenic pulmonary edema and one with diffuse alveolar bleeding.

**RESULTS:**

Eleven of the patients (74%) were at the weaning period at the time of unplanned extubation. Among these 11 patients, NPPV was successful in 10 (91%) and only one (9%) was reintubated due to the failure of NPPV. The remaining four patients (26%) had pneumonia and none of them were at the weaning period at the time of extubation, but their requirement for mechanical ventilation was gradually decreasing. Unfortunately, an NPPV attempt for 6–8 h failed and these patients were reintubated.

**CONCLUSIONS:**

Patients with unplanned extubation before the weaning criteria are met should be intubated immediately. On the other hand, when extubation develops during the weaning period, NPPV may be an alternative. The present study was conducted with a small number of patients, and larger studies on the effectiveness of NPPV in unplanned extubation are warranted for firm conclusions.

The frequency of unplanned extubation ranges between 3 and 16% among patients on mechanical ventilatory support.[1] About 55% of patients with unplanned extubation require reintubation; however, this figure decreases to 30% if the extubation occurs during the weaning period.[2]

Invasive mechanical ventilation is a safe method in intensive care unit (ICU) patients when noninvasive positive pressure ventilation (NPPV) is contraindicated or not applicable. However, complications like nosocomial pneumonia and intubation-related complications may occur.[3–7] Prevalence of nosocomial pneumonia rises significantly with recurrent intubations.[8] Increases in mortality, duration of hospitalization, duration of ICU stay and need for chronic care have been reported in patients requiring reintubation following an unsuccessful extubation attempt.[9]

Therefore, early administration of NPPV before the development of respiratory failure may decrease the frequency of reintubations, which was demonstrated in previous studies, along with a decrease in ICU mortality.[10,11]

The aim of the present study was to examine the effect of NPPV following an unplanned extubation on the prevalence of reintubation.

## Materials and Methods

A total of 15 patients were included among 130 intubated patients receiving mechanical ventilation at our hospital during the year 2004. Our ICU has seven beds and during the daytime, one specialist physician, two residents and two nurses are present for seven patients. During night shifts, two residents and one nurse are present. Intubation tube is fixed by a cotton bandage. Patients are sedated using the Ramsay sedation scale.

In our institution, weaning is performed according to standard protocols either by gradually decreasing pressure support or once daily administration of T-tube trail.[12,13] Following the disappearance of factor(s) leading to the need for mechanical ventilation, patients undergo daily evaluation for the possibility of weaning and eligible patients are extubated. Together with the improvement of the underlying condition, all of the following criteria should be met for T-tube trail or a gradual decrease of pressure support: normal consciousness, no hypothermia or sepsis, PaO_2_ > 60 when FiO_2_ < 40%, no cardiac ischemia or arrhythmia and cuff leak volume > 110 ml.[14] According to previously published criteria, patients tolerating T-tube trail are extubated.

Patients with the following conditions were excluded from the study: coma, excess pulmonary secretions and inability to protect airways, agitation or lack of coordination, anatomical deformity preventing the application of face mask, uncontrolled cardiac ischemia or arrhythmia and failure of more than two organs.

A need for reintubation within 72 h following unplanned extubation was considered as failure. The decision of either reintubation or NPPV administration following unplanned extubation was made by the responsible physician of the ICU. Conventional intensive care monitors were used throughout the study (Puritan-Bennett, 7200, Carlsbad, CA, USA). All patients had been receiving mechanical ventilation at pressure support ventilation mode prior to unplanned extubation.

Respiratory or cardiac arrest, a hold of respiration together with unconsciousness, agitation that cannot be controlled by sedation, massive aspiration, persistent failure to expectorate the secretions, a heart rate < 50 accompanied by a deterioration of consciousness and hemodynamic instability unresponsive to the administration of fluids and vasoactive agents were considered as indications for emergency intubation.[15] After extubation, they initially received support with the same pressure levels prior to unplanned extubation and the following titrations were made: respiratory rate < 25/min on pressure support, oxygen saturation > 92% and pH > 7.35.

NPPV was initiated in patients without an indication for emergency intubation. Patients were reintubated due to the failure of NPPV if they met at least one of the following criteria: an increase in PaCO_2_ ≥ 10 mm Hg and decrease in pH ≥ 0.10; PaO_2_ < 60 mm Hg or SaO_2_ < 90% despite the use of a high fraction of inspired oxygen; tachypnea, use of accessory respiratory muscles, thoracoabdominal paradox; inability to protect the airway, excess pulmonary secretions, changes in mental status, unable to tolerate NPPV.[16,17] NPPV was interrupted every 4 h for daily facial and oral care of the patients. Arterial blood gases were measured 1 h after the initiation of NPPV and repeated twice daily and when needed. Respiratory pattern, consciousness and vital signs were continuously monitored and the APACHE II score was recorded at admission to the ICU.

The data were analyzed by SPSS Inc., 233 S. Wacker Drive, 11^th^ floor, Chicago, IL 60606-6307 (14.0 for Windows) statistical software. Results are expressed as means ± SD . The Mann–Whitney *U*-test was used for comparison of the groups.

A *P*-value of < 0.05 was considered significant.

## Results

Patient data are depicted in Table 1. A total of 130 intubated patients receiving mechanical ventilation at our ICU were included in our study and unplanned extubation occurred in 15 (11%) of them (12 males, age: 57 ± 24 years, APACHE II score: 19 ± 7). All patients received mechanical ventilatory support for more than 3 days. Indications for intubation and mechanical ventilation were as follows: pneumonia (60%), epilepsy (20%), cardiogenic pulmonary edema (6.6%), hemodynamic stability due to gastrointestinal bleeding followed by unconsciousness (6.6%) and alveolar hemorrhage (6.6%). In 11 patients, weaning criteria were being explored at the time of unplanned extubation. In the remaining four patients, the need for mechanical ventilation was still present despite an improvement in the underlying condition. All patients were receiving mechanical ventilatory support at pressure support ventilation mode before the unplanned extubation.

**Table 1:** Baseline characteristics of patients

	NPPV successful	NPPV unsuccessful
Patients (n)	10	5
Age	51 ± 26	61 ± 19
APACHE II score	19 ± 8	19 ± 4
Pneumonia	4	5
Epilepsy	3	0
Alveolar hemorrhage	1	0
GIS bleeding	1	0
Cardiogenic pulmonary edema	1	0

Of the 11 patients at the weaning period, one (9%) was reintubated due to the failure of NPPV. All of the remaining four patients who had not achieved weaning period (all were admitted for pneumonia) failed to respond to the 6–8 h trial of NPPV and were reintubated.

Table 2 shows the arterial blood gas measurements following unplanned extubation. Arterial blood gases and APACHE II scores were similar in patients responding and not responding to NPPV (*P* > 0.05 for all comparisons).

**Table 2:** Physiological parameters following unplanned extubation

	pH	PCO_2_	HCO_3_	PO_2_/FiO_2_
Failure	7.42 ± 0.01	39.5 ± 10.97	25.17 ± 6.37	281.8 ± 44.81
Success	7.40 ± 0.05	41.45 ± 7.78	24.91 ± 3.65	272.73 ± 58.55
*P*	0.35	0.7	0.93	0.72

None of the four patients in the unsuccessful group complied with NPPV and all were agitated during the treatment. Only one patient had difficulty in the expectoration of the secretions in the successful group compared with four patients in the group unresponsive to NPPV treatment.

## Discussion

The findings of the present observational study suggest that NPPV may be an option in patients with unplanned extubation while on mechanical ventilatory support at the ICU if the patient is at the stage where weaning criteria are being explored. However, patients should be examined for the presence of contraindications before NPPV administration. On the other hand, patients who do not meet weaning criteria should be intubated without delay in case unplanned extubation develops.

Unplanned extubation is quite common in ICU patients receiving mechanical ventilatory support (3–16%).[1] Among its main causes are inadequate sedation and insufficient nursing care during positioning. Nevertheless, unplanned extubation may develop under optimal conditions where sedation is adequate and all necessary precautions are taken.[18] Unplanned extubation was developed in 11% of our patients during 1 year of follow-up, which is in the range of previously reported rates. Patients are routinely sedated in our unit and the Ramsay sedation scale levels are kept between 3 and 4.

About 55% of the patients with unplanned extubation finally require reintubation. Although the frequency of reintubation is less among patients at the period when weaning criteria are being explored, a 30% rate has been reported in a previous study.[2] The rate of reintubation was 9% in our study when NPPV was used following unplanned extubation developed at the time when weaning criteria were being explored.

Reintubation increases the prevalence of infection at the ICU unit. In addition, it prolongs the duration of mechanical ventilation. NPPV is able to improve respiratory failure and decrease mortality and duration of hospitalization without intubation in eligible patients. Several studies investigated the use of NPPV in failure of planned extubation or in case of unplanned extubation. NPPV is the first treatment of choice in acute respiratory failure secondary to chronic obstructive pulmonary disease and in cardiogenic acute pulmonary edema.[19] However, these effects do not result in improved rates of survival and a higher number of adverse events occurred with NPPV treatment.[20,21] Particularly, NPPV is recommended in immunosuppressive patients with acute respiratory failure as it decreases infectious complications caused by intubation. Data regarding the successful use of NPPV in patients with hypoxemic respiratory failure are growing.[22]

Jiang *et al*, randomized 93 patients who developed unplanned extubation or failed to be extubated into oxygen treatment or NPPV. They did not find any significant difference between the two groups in terms of reintubation frequency. However, that study included patients developing unplanned extubation and patients failed to be extubated in the same group.[23]

Most of the studies exploring the effect of NPPV on the frequency of reintubation are conducted on patients failing extubation. Keenan *et al*, failed to demonstrate the superiority of NPPV over standard treatment in their study with patients developing respiratory stress within the 48 h following extubation.[24] Esteban *et al*, conducted a study in 221 patients comparing NPPV and standard treatment and found that NPPV did not reduce the need for reintubation.[16]

Most importantly, this study showed that delaying intubation in case of acute respiratory failure occurring after extubation was associated with increased mortality. Thus, in case of unplanned extubation, the patients must be carefully followed-up, NPPV contraindications should be rapidly reviewed and patients not eligible for NPPV should be reintubated without delay.

In order to evaluate the effectiveness of NPPV in preventing the need for intubation, Jiang *et al*, initiated NPPV following extubation before the development of respiratory distress and found that NPPV did not result in any survival benefit compared with standard medical treatment.

Our study differs from other studies in that our patients had an unplanned extubation without a schedule. Although most of them were at a period when weaning criteria were being explored, their evaluation within the last 24 h did not permit extubation, i.e. weaning criteria were not completely met.

Of the 11 patients at the weaning period, 10 responded satisfactorily to NPPV treatment. Indication for intubation had been pneumonia in four of the patients with successful response. However, evaluation performed just after unplanned extubation revealed minimum amounts of secretions. Among main factors effecting NPPV success are copious amounts of secretions at the airways and/or difficulty in expectorating these secretions due to muscular weakness. The good cooperation and compliance of patient group to NPPV treatment might have contributed to this success.

In the successful group, two patients were initially intubated for epilepsy and one for the protection of airways during hemodynamic shock and unconsciousness secondary to gastrointestinal bleeding. By chance, mechanical ventilation had not been complicated by infection in these patients and NPPV was administered after unplanned extubation following more than 72 h of mechanical ventilation.

In two patients in the weaning period, the amount of secretions was increased. One of the patients did not respond to NPPV treatment and was reintubated. Although the number of our patients is low, considering the previous studies and the present results, it suggests that presence of secretions is one of the most important factors for NPPV success. Also, loss of muscular strength for aiding the expectoration of secretions may also be an important factor. These patients also had a compliance problem with NPPV and were agitated.

There are two limitations of the present study. It has limited number of patients and it is an observational study. Therefore, to draw firm conclusions on the effect of NPPV in patients with unplanned extubation, randomized studies with a larger number of patients are warranted.

## Conclusion

In conclusion, NPPV may be an option in patients with unplanned extubation during the weaning period. However, when the unplanned extubation occurs before weaning criteria are met, NPPV contraindications should be rapidly reviewed and patients not eligible for NPPV should be reintubated without delay.
